# Nonconvex Nonlocal Tucker Decomposition for 3D Medical Image Super-Resolution

**DOI:** 10.3389/fninf.2022.880301

**Published:** 2022-04-25

**Authors:** Huidi Jia, Xi'ai Chen, Zhi Han, Baichen Liu, Tianhui Wen, Yandong Tang

**Affiliations:** ^1^State Key Laboratory of Robotics, Shenyang Institute of Automation, Chinese Academy of Sciences, Shenyang, China; ^2^Institutes for Robotics and Intelligent Manufacturing, Chinese Academy of Sciences, Shenyang, China; ^3^University of Chinese Academy of Sciences, Beijing, China; ^4^School of Professional Studies, Columbia University, New York, NY, United States

**Keywords:** 3D super-resolution, low rank tensor decomposition, nonlocal self-similarity, 3D total variation, medical image

## Abstract

Limited by hardware conditions, imaging devices, transmission efficiency, and other factors, high-resolution (HR) images cannot be obtained directly in clinical settings. It is expected to obtain HR images from low-resolution (LR) images for more detailed information. In this article, we propose a novel super-resolution model for single 3D medical images. In our model, nonlocal low-rank tensor Tucker decomposition is applied to exploit the nonlocal self-similarity prior knowledge of data. Different from the existing methods that use a convex optimization for tensor Tucker decomposition, we use a tensor folded-concave penalty to approximate a nonlocal low-rank tensor. Weighted 3D total variation (TV) is used to maintain the local smoothness across different dimensions. Extensive experiments show that our method outperforms some state-of-the-art (SOTA) methods on different kinds of medical images, including MRI data of the brain and prostate and CT data of the abdominal and dental.

## 1. Introduction

High-resolution (HR) medical images provide rich detailed information that is critical for accurate lesion segmentation, diagnosis, and treatment (Greenspan, 2009; Shi et al., 2010). Currently, the most widely used imaging techniques in clinical settings and research include magnetic resonance imaging (MRI) and computed tomography (CT). However, both MRI and CT have their limitations. The main limitation of MRI is the balance between image quality and scan time (Bustin et al., 2018). In general, it requires a long acquisition time to obtain an HR MRI with a high signal-to-noise ratio (SNR). This not only costs a lot but also affects patients' breathing and causes motion artifacts. Contrast-enhanced CT can show small lesions more clearly and blood flow in lesions. Due to the influence of a high radiation dose and contrast agent, contrast-enhanced CT scans are not allowed in many cases, such as in patients with hyperthyroidism and hypersensitivity (Marcelino et al., 2019). For instance, micro-CT (μCT), which is applied to determine the 3D structure of teeth, has a higher resolution than cone beam computed tomography (CBCT), but it can only be used for extracted teeth due to the acquisition time and radiation (Hatvani et al., 2019). Super-resolution provides an efficient method for obtaining HR images from low-resolution (LR) images when acquisition conditions are limited. Therefore, super-resolution has become an important research issue in image processing and is widely applied in medical imaging (Zhang et al., 2012b; Shi et al., 2015; Hatvani et al., 2019; Qiu et al., 2021; Zhao et al., 2021; Zhu and Qiu, 2021).

Super-resolution image reconstruction is a highly ill-posed problem because it predicts multiple HR images from a given LR image. To solve this ill-posed problem, various super-resolution methods have been proposed. The most direct methods are based on interpolation (Duchon, 1979; Keys, 1981), such as nearest neighbor (NN) interpolation, bilinear interpolation, and bicubic interpolation. Interpolation-based methods are fast but not very accurate. Learning-based methods (Zhang et al., 2012a; Salvador, 2016; Lim et al., 2017; Liu et al., 2017) learn example patches from fixed and finite HR image sets. Thus, the performance of these methods highly depends on the learned HR image patches. Deep learning-based methods (Ledig et al., 2017; Zhao et al., 2019; Fan et al., 2020; Liu et al., 2021) have obtained outstanding performances in high-level image processing tasks, such as alignment, segmentation, and object detection. Recently, they also began to show their advantage in low-level image processing tasks. It is widely known that sufficient data are necessary for training an effective deep learning model. However, unlike natural images, medical databases for training are not always available due to some privacy regulations and laws.

As an important and powerful modeling tool, low-rank has attracted increasing attention in many fields, such as signal processing (E-Asim et al., 2020), image processing (Xu et al., 2015; Chen et al., 2020), and machine learning (Yi et al., 2021). Adding low-rank regularization can achieve a good reconstruction result in super-resolution (Shi et al., 2015; Yair and Michaeli, 2018). Liu et al. (2016) exploited the low-rank property of an image by reshaping multidimensional data to a matrix and applying a low-rank constraint to the matrix. Veganzones et al. (2016) took advantage of the low-rank property to propose two HSI super-resolution methods by local dictionary learning using end member induction algorithms. Although these low-rank matrix methods obtain good super-resolution results, they ignore considerable structural information of data in the process of reshaping an image to a 2D matrix.

Mathematically, MRI and CT images are multidimensional data with high spatial resolution and slice resolution. Tensors provide a natural way to represent multidimensional data. A tensor is a multiway array that can be viewed as a generalization of vectors and matrixes. For 3D medical images, there is a strong correlation between slices, and a 3rd-order tensor can be used to represent an image to avoid destroying the process of reshaping data into the matrix. It has been proven that tensors are a reasonable representation that can preserve the original structure of data and significantly improve the quality of reconstruction images (Liu et al., 2013; Yin et al., 2017; Li et al., 2018; Xie et al., 2018; Hatvani et al., 2019; Prevost et al., 2019). According to different decompositions of tensor, a series of low-rank tensor methods have emerged. Liu et al. (2013) extended low-rank matrix completion to the tensor case for tensor completion. Different from the matrix, the rank of a tensor is not clearly defined and the decomposition of the tensor is not unique. The CP (CANDECOMP/PARAFAC) decomposition (Carroll and Chang, 1970) of a tensor is a representation based on a sum of several rank-1 tensors. CP decomposition based methods require a small memory space, but these methods need to predefine the rank, and the calculation of CP rank is an NP-hard problem (Kolda and Bader, 2009). Tucker decomposition (Tucker, 1966) represents a tensor as the product of a core tensor and several factor matrixes and minimizes the rank of the core tensor and the factor matrixes. The appearance of Tucker decomposition solves the calculation problem of CP rank. Previous methods (Chen et al., 2014; Dian et al., 2017) have proved that Tucker decomposition was effective and obtained satisfactory results in many fields. Li et al. (2017) applied tensor Tucker decomposition to the tensor completion problem and utilized the trace norm as a low-rank constraint to the factors of Tucker decomposition (Tucker, 1966). In low-rank structure learning, tensor norms, e.g., trace norms and nuclear norms, penalize large entries of vectors overly and usually introduce modeling bias (Leng et al., 2006). To correct the estimation bias of the convex tensor norms, unbiased folded-concave norms are considered. Hatvani et al. (2019) proposed a tensor factorization method for 3D super-resolution and applied it to dental CT. However, they did not consider the prior information of images.

As there are often many similar structures in human organs or tissues, we consider nonlocal self-similarity in our model. Nonlocal self-similarity is an important patch-based prior. This means that for a given patch in an image, some similar patches within the whole image can be found. In many tasks, such as denoising and recovery, nonlocal similarity-based methods (Dabov et al., 2007; Mairal et al., 2009; Wang et al., 2018) have demonstrated their effectiveness. Most Tucker decomposition-based methods (Chen et al., 2014; Dian et al., 2017; Li et al., 2017; Yair and Michaeli, 2018) only employ the global prior information in their models. To further exploit the low-rank prior hidden in the data and improve the reconstruction performance of super-resolution, nonlocal similarity in the tensor cubes is exploited.

Due to statistical uncertainty in physical measurements, inevitable noise is introduced in MRI and CT data. The noise in medical images will affect the clinical diagnosis accuracy and increase the difficulty of high-level tasks such as registration and segmentation (Zhang et al., 2015; Diwakar and Kumar, 2018). TV, which is defined as the integral of the absolute gradients of the image, is an effect regularization for suppressing noise and preserving the local spatial consistency of images. Shi et al. (2015) combined both global low-rank priori and two-dimensional TV regularization to obtain a higher resolution MR image. The two-dimensional TV only considers the local smoothness without utilizing the interframe smoothness. To take advantage of the spatial and interframe local smoothness simultaneously, weighted 3-dimensional TV (3D TV) is adopted in super-resolution reconstruction.

**Figure 1:**
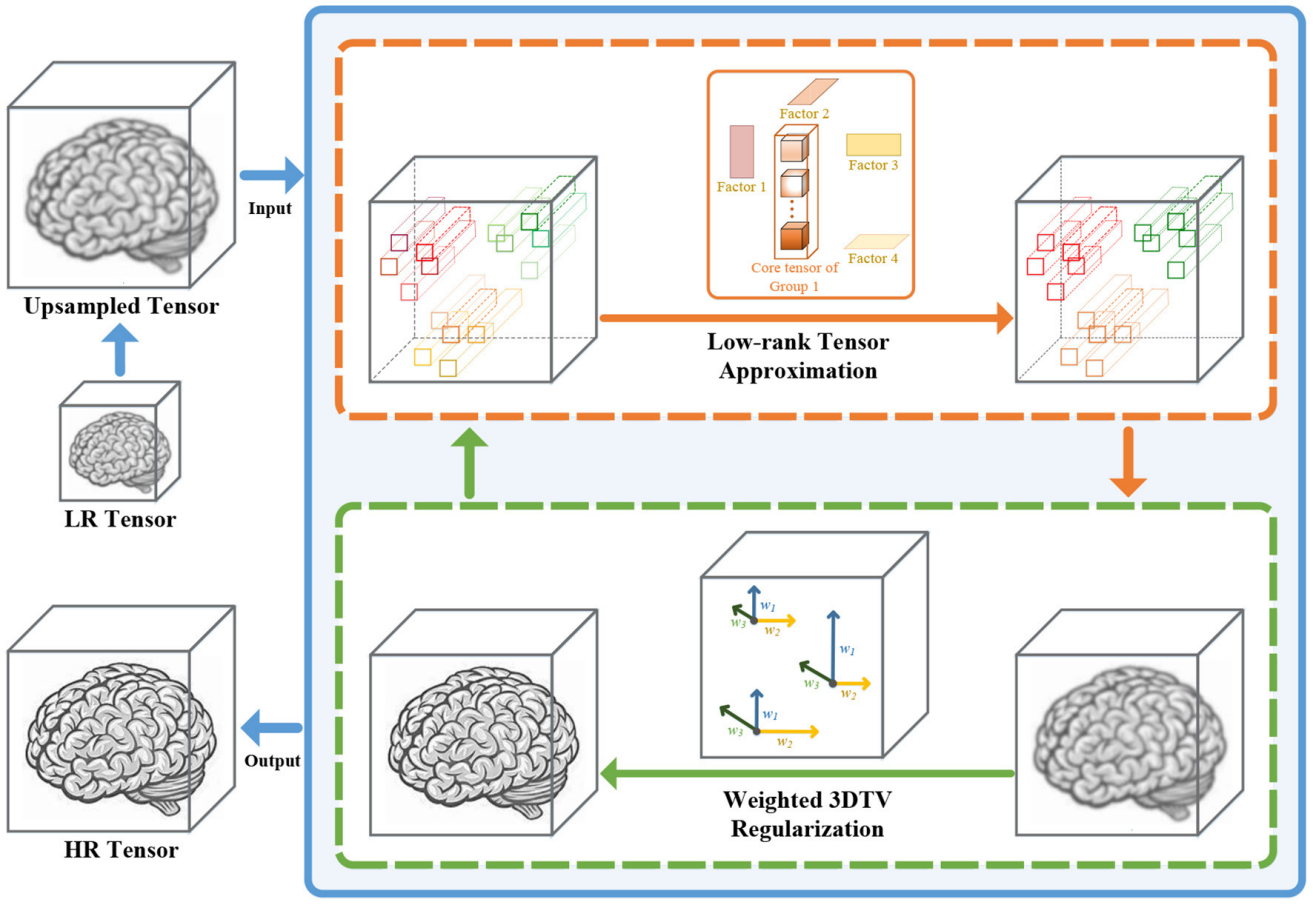
The detailed framework of our method. The proposed method is divided into two parts, the low-rank regularization term, and the 3D total variation (TV) regularization term. The low-rank regularization term (the orange box) reconstructs the main information of the data by performing Tucker decomposition for the nonlocal similar self-similarity patches. The 3D TV regularization term (the green box) uses the local smoothness of the data to suppress the noise and keep the details.

In this article, we propose a super-resolution method for 3D medical images based on nonconvex nonlocal Tucker decomposition with weighted 3D total variation (NNTDTV). Different from most existing single image super-resolution methods, our method improves volume super-resolution, i.e., it not only improves the spatial resolution of images but also improves the slice resolution. The framework of our method is shown in Figure 1. The main contributions of this article are summarized as follows:

1. We propose a nonconvex nonlocal Tucker decomposition-based super-resolution method to approximate HR medical images by utilizing the nonlocal similarity structure hidden in 3D medical images. A nonconvex optimization procedure is used to avoid estimation bias caused by the traditional convex procedure.
2. Weighted 3D total variation (TV) is used to exploit the smoothness of 3D medical images and suppress noise to some extent.
3. Extensive experiments show that, compared with the existing methods, our method has better performance and generality on different kinds of medical images. The reconstructed super-resolution images by our model have a clearer edge while maintaining local smoothness.

## 2. Methods

### 2.1. Notation and Preliminaries

A tensor is a multidimensional array that can be viewed as a high-dimensional generalization of a matrix. We consider scalars as zero-order tensors and denote them by lowercase letters (*x, y, z*, ⋯ ). Vectors and matrices are first-order and second-order tensors and denoted by bold lowercase letters (**x**, **y**, **z**, ⋯ ) and uppercase letters (*X, Y, Z*, ⋯ ), respectively. A high-order tensor can be expressed as $X\in {\mathbb{R}}^{I_{1}\times I_{2}\times \cdots \times I_{N}}$, and its element is *x*_*i*_1_, *i*_2_, ⋯ , *i*_*N*__. The mode-n matricization or unfolding of a tensor $X\in {\mathbb{R}}^{I_{1}\times I_{2}\times \cdots \times I_{N}}$ is the operation of reshaping a tensor into a matrix $X_{\left(n\right)}\in {\mathbb{R}}^{I_{n}\times \left(I_{1}\cdots I_{n-1}I_{n+1}\cdots I_{N}\right)}$. The elements (*i*_1_, …, *i*_*n*−1_, *i*_*n*_, *i*_*n*+1_, …*i*_*N*_) of matrix *X*_(*n*)_ satisfy:


(1) $$\begin{array}{l} j=\text{1}+\overset{N}{\underset{k=1,k\neq n}{\sum }}\left(i_{k}-1\right)J_{k}\text{ with }J_{k}=\overset{k-1}{\underset{m=1,m\neq n}{\prod }}I_{m}. \end{array}$$

For two tensors of the same size, their inner product is defined as:


(2) $$\begin{array}{l} \langle X,Y\rangle :=\underset{i_{1},i_{2},\ldots ,i_{K}}{\sum }x_{i_{1},i_{2},\ldots ,i_{K}}\cdot y_{i_{1},i_{2},\ldots ,i_{K}}. \end{array}$$

The corresponding Frobenius norm is defined as:


(3) $$\begin{array}{l} ||X|{|}_{F}=\sqrt{\langle X,X\rangle }. \end{array}$$

A tensor $X\in {\mathbb{R}}^{I_{1}\times I_{2}\times \cdots \times I_{N}}$ and a matrix $Z\in {\mathbb{R}}^{J\times I_{n}}$ 's mode-*n* product is:


(4) $$\begin{array}{l} Y=X\times_{n}Z\in {\mathbb{R}}^{I_{1}\times \cdots I_{n-1}\times J\times I_{n+1}\cdots \times I_{n}}, \end{array}$$

with entries


(5) $$\begin{array}{l} y_{i_{1},\cdots \;,i_{n-1},j,i_{n+1},\cdots N}=\overset{I_{n}}{\underset{i_{n}=1}{\sum }}\left(x_{i_{1},\cdots \;,x_{N}}z_{j,i_{n}}\right). \end{array}$$

For a tensor $X\in {\mathbb{R}}^{I_{1}\times I_{2}\times \cdots \times I_{N}}$, its Tucker decomposition is defined as:


(6) $$\begin{array}{l} \begin{array}{c} X=\overset{S_{1}}{\underset{s_{1}=1}{\sum }}\times \overset{S_{N}}{\underset{s_{N}}{\sum }}g_{s_{1}s_{2}\ldots s_{N}}\left[u_{s_{1}}^{\left(1\right)}◦u_{s_{2}}^{\left(2\right)}◦\cdots ◦u_{s_{N}}^{\left(N\right)}\right]\\ =G\times_{1}U^{\left(1\right)}\times_{2}U^{\left(2\right)}\cdots \times_{N}U^{\left(N\right)}. \end{array} \end{array}$$

where $G\in {\mathbb{R}}^{S_{1}\times S_{2}\times \cdots \times S_{N}}$ denotes the core tensor and ${\left\{U^{\left(n\right)}\right\}}_{n=1}^{N}\in {\mathbb{R}}^{I_{n}\times {\text{S}}_{n}}$ denote the decomposition factors by Tucker decomposition of the tensor.

### 2.2. Problem Formulation

Affected by acquisition modality, motion blur, and noise, the observation model of an LR image can be expressed as:


(7) $$\begin{array}{l} Y=DSX+\varepsilon , \end{array}$$

where $Y\in {\mathbb{R}}^{w\times h\times S}$ denotes the observed LR image, D denotes the downsampling operator, S denotes the blurring operator, ε is the observation noise, and $X\in {\mathbb{R}}^{W\times H\times S}$ is the HR image that we want to reconstruct. To reconstruct an HR image, we can estimate X by minimizing


(8) $$\begin{array}{l} \underset{X}{\text{min }}||DSX-Y|{|}_{F}^{2}. \end{array}$$

According to Equation (8), recovering HR X from Y is an ill-posed inverse problem. This problem can be solved by introducing some regularization terms. Thus, we obtain the following cost function:


(9) $$\begin{array}{l} \underset{X}{\text{min }}||DSX-Y|{|}_{F}^{2}+\lambda R\left(X\right), \end{array}$$

where $||DSX-Y|{|}_{F}^{2}$ is the fidelity term, R(X) is the regularization term, and λ is a parameter used to balance the fidelity term and regularization term. The reconstruction effectiveness of the proposed model is closely related to the rationality of regularization terms.

### 2.3. Proposed Model

We use Weighted 3D TV and nonlocal low-rank terms as the regularization terms to approximate X in our model. Therefore, the proposed model for the super-resolution task is formulated as follows:


(10) $$\begin{array}{l} \underset{X}{\text{min }}||DSX-Y|{|}_{F}^{2}+\lambda_{tv}||X|{|}_{3\text{DTV}}+\lambda_{\text{rank}}rank\left(P_{X}^{k}\right), \end{array}$$

where ||·||_3*DTV*_ is the 3D TV regularization and *rank*(·) denotes the low-rank terms. Here, $P_{X}^{k}$ denotes the *k*th group of patches, *k* ∈ [1, *K*] and *K* is the number of groups, and the order of $P_{X}^{k}$ is *N*. λ_*tv*_ and λ_*rank*_ are the regularization parameters.

#### 2.3.1. 3DTV Regularization

Total variation is a well-known regularization approach for preserving the local spatial consistency of data in various image processing tasks. Traditional TV is designed to exploit the local spatial smoothness prior to removing noise and retaining edges in images. Considering that 3D medical images can be seen as a 3rd-order tensor, smoothness is in three dimensions. Therefore, we introduce the weighted 3D TV to model the spatial and slice smoothness simultaneously. 3D TV is formulated as follows:


(11) $$\begin{array}{l} \begin{array}{c} TV\left(X\right)=\underset{ijk}{\sum }w_{1}|x_{ijk}-x_{ij,k-1}|+w_{2}|x_{ijk}-x_{i,j-1,k}|\\ +w_{3}|x_{ijk}-x_{i-1,jk}| \end{array} \end{array}$$

where *x*_*ijk*_ denotes the pixel at location (*i, j*) in the *k*th band, and *w*_*d*_(*d* = 1, 2, 3) denotes the weight along with different modes of X. With constraints on spatial and slice dimensions, this weighted 3D TV remains piecewise smoothness in three dimensions.

#### 2.3.2. Nonlocal Low-Rank Regularization

The spatial non-local similarity is one of the most important priors in image processing. For a given local patch in an image, we can find some other similar patches. To exploit this prior, we perform a series of operations on the original tensor. First, we separate image X into a set of patches $\Omega ={\left\{P_{k}\in {\mathbb{R}}^{b\times b\times B}\right\}}_{k=1}^{K}$, where *b* × *b* is the size of the patch, *B* is the number of bands, and *K* is the number of patches with overlap. Second, for a given local patch, we find *d*-1 patches with the smallest Euclidean distance from it. Then, we stack them together with a local patch, forming a 4th-order tensor $P_{X}^{k}$ with size *b* × *b* × *B* × *d*.

The global spectral correlation and spatial nonlocal similarity of images give the matched 4th-order tensor $P_{X}^{k}$ a good low-rank property. Furthermore, we perform Tucker decomposition for the nonlocal low-rank terms $P_{X}^{k}$ to exploit the low-rank characteristics and obtain:


(12) $$\begin{array}{l} \begin{array}{c} \underset{U_{k}^{\left(n\right)},G_{k}}{\text{min }}\lambda_{1}\overset{K}{\underset{k=1}{\sum }}\overset{N}{\underset{n=1}{\sum }}{\left||U_{k}^{\left(n\right)}|\right|}_{P_{\lambda }}\text{+}\lambda_{2}\overset{K}{\underset{k=1}{\sum }}||G_{k}|{|}_{F}^{2}\;s.t.P_{X}^{k}\\ =G_{k}\times_{1}U_{k}^{\left(1\right)}\times_{2}U_{k}^{\left(2\right)}\cdots \times_{4}U_{k}^{\left(N\right)}, \end{array} \end{array}$$

where $G_{k}$ and $U_{k}^{\left(n\right)}$ are the core tensor and decomposition factors, respectively, obtained by Tucker decomposition of $P_{X}^{k}$, and λ_1_ and λ_2_ are the regularization parameters.

The fold-concave penalty is a nonconvex norm. Different from the matrix nuclear norm that punishes the large singular value resulting in bias (Leng et al., 2006), the fold-concave penalty is theoretically proven to be an almost unbiased estimation of the rank (Fan and Li, 2001). For a given matrix *X*, we define its folded-concave norm as:


(13) $$\begin{array}{l} ||X|{|}_{P_{\lambda }}:=\sum_{j=1}^{r}P_{\lambda }\left[\sigma_{j}\left(X\right)\right], \end{array}$$

where *r* is the rank of *X*, σ_*j*_(*X*) is its *j*th singular value, and *P*_λ_ is called the folded-concave penalty function.

The minmax concave plus (MCP) penalty is one of the fold-concave penalties, and it has a simple form and excellent performance. In this article, we use the MCP penalty to exploit the low-rank structure of the Tucker decomposition factors $U_{k}^{\left(n\right)}\left(n=1,2,\cdots \;,N\right)$ of $P_{X}^{k}$. MCP penalty defined as follows:


(14) $$P_{\lambda }(t)=\{\begin{array}{ll} a\lambda^{2}/2, & \text{if }\left|t\right|\geq a\lambda ,\\ \lambda \left|t\right|-\frac{t^{2}}{2a}, & \text{otherwise}, \end{array}$$

where *a* is a constant. In addition, the last term in Equation (12) imposes the Frobenius norm on the core tensor $G_{k}$ to avoid overfitting.

In summary, our tensor super-resolution model is formulated as follows:


(15) $$\begin{array}{l} \begin{array}{c} \underset{X}{\text{min }}||DSX-Y|{|}^{2}+\lambda_{tv}||X|{|}_{3\text{DTV}}+\lambda_{1}\overset{K}{\underset{k=1}{\sum }}\overset{N}{\underset{n=1}{\sum }}{\left||U_{k}^{\left(n\right)}|\right|}_{P_{\lambda }}\\ +\lambda_{2}\overset{K}{\underset{k=1}{\sum }}||G_{k}|{|}_{F}^{2},\\ s.t.P_{X}^{k}=G_{k}\times_{1}U_{k}^{\left(1\right)}\times_{2}U_{k}^{\left(2\right)}\cdots \times_{4}U_{k}^{\left(N\right)}. \end{array} \end{array}$$

### 2.4. Optimization

For optimization purposes, we rewrite Equation (15) as:


(16) $$\begin{array}{l} \begin{array}{c} \underset{X}{\text{min }}||DSX-Y|{|}^{2}+\lambda_{tv}||G_{w}\left(X\right)|{|}_{1}+\lambda_{1}\overset{K}{\underset{k=1}{\sum }}\overset{N}{\underset{n=1}{\sum }}{\left||U_{k}^{\left(n\right)}|\right|}_{P_{\lambda }}\\ \;+\lambda_{2}\overset{K}{\underset{k=1}{\sum }}||G_{k}|{|}_{F}^{2},\\ \;s.t.P_{X}^{k}=G_{k}\times_{1}U_{k}^{\left(1\right)}\times_{2}U_{k}^{\left(2\right)}\cdots \times_{4}U_{k}^{\left(N\right)}, \end{array} \end{array}$$

where $||G_{w}\left(X\right)|{|}_{1}=\left[w_{1}\times G_{1}\left(\cdot \right);w_{2}\times G_{2}\left(\cdot \right);w_{3}\times G_{3}\left(\cdot \right)\right]$ is the weighted three-dimensional difference operator and *G*_1_, *G*_2_, and *G*_3_ are the first-order difference operators in three dimensions.

We introduce some necessary auxiliary variables, including X=M and $F=G_{w}X$, to split the interdependencies of the terms in Equation (16) and obtain:


(17) $$\begin{array}{l} \underset{X}{\min}{\left\|DSX-Y\right\|}^{2}+\lambda_{tv}{\left\|G_{w}(X)\right\|}_{1}+\lambda_{1}\sum_{k=1}^{K}\sum_{n=1}^{N}{\left\|U_{k}^{\left(n\right)}\right\|}_{P_{\lambda }}\\ +\lambda_{2}\sum_{k=1}^{K}{\left\|G_{k}\right\|}_{F}^{2},\\ s.t.\;P_{X}^{k}=G_{k}\times_{1}U_{k}^{\left(1\right)}\times_{2}U_{k}^{\left(2\right)}\cdots \times_{4}U_{k}^{\left(N\right)},\;X=ℳ,\\ ℱ=G_{w}X,\\ {\left\{V_{k}^{\left(n\right)}=U_{k}^{\left(n\right)}\right\}}_{n=1\;K=1}^{N\;K},\;\{P_{ℳ}^{k}=G_{k}\times_{1}U_{k}^{\left(1\right)}\times_{2}U_{k}^{\left(2\right)}\cdots \\ \times_{N}U_{k}^{\left(N\right)}{\}}_{k=1}^{K} \end{array}$$

We solve the cost function Equation (17) by adopting the alternating direction method of multipliers (ADMM) algorithm and obtain the object function as follows:


(18) $$\begin{array}{l} \begin{array}{c} L\left(X,F,U_{k}^{\left(n\right)},V_{k}^{\left(n\right)},G_{k},M,W_{1},\ldots ,W_{4}\right)\\ =||DSX-Y|{|}_{F}^{2}\text{+}\frac{w_{1}}{2}||X-M+\frac{W_{1}}{w_{1}}|{|}_{F}^{2}+\lambda_{tv}||F|{|}_{1}\\ +\frac{w_{2}}{2}||F-G_{w}X+\frac{W_{2}}{w_{2}}|{|}_{F}^{2}\\ +\overset{K}{\underset{k=1}{\sum }}\left[\lambda_{2}||G_{k}|{|}_{F}^{2}+\overset{N}{\underset{n=1}{\sum }}\left(\lambda_{1}{\left||U_{k}^{\left(n\right)}|\right|}_{P_{\lambda }}\right)\right]\\ +\overset{K}{\underset{k\;=\;1}{\sum }}\overset{N}{\underset{n\;=\;1}{\sum }}\left(\frac{w_{3}}{2}||U_{k}^{\left(n\right)}-V_{k}^{\left(n\right)}+\frac{{W_{3}}_{\left(k\right)}^{n}}{w_{3}}|{|}_{F}^{2}\right)\\ +\overset{K}{\underset{k=1}{\sum }}\left(\frac{w_{4}}{2}||P_{M}^{k}-G_{k}\times_{1}V_{k}^{\left(1\right)}\times_{2}V_{k}^{\left(2\right)}\cdots \times_{N}V_{k}^{\left(N\right)}+\frac{{W_{4}}_{\left(k\right)}}{w_{4}}|{|}_{F}^{2}\right) \end{array} \end{array}$$

where $W_{1}$, $W_{2}$, ${{\left\{{W_{3}}_{k}^{\left(n\right)}\right\}}_{n=1}^{N}}_{K=1}^{K}$, and ${\left\{{W_{4}}_{\left(k\right)}\right\}}_{k=1}^{K}$ are Lagrange multipliers. According to ADMM, we divide Equation (18) into Equation (6) subproblems and solve them by iteratively updating the variables. The following are the detailed variables updating procedures. The optimization procedure of the proposed model is shown in Algorithm 1.

**Algorithm 1:**
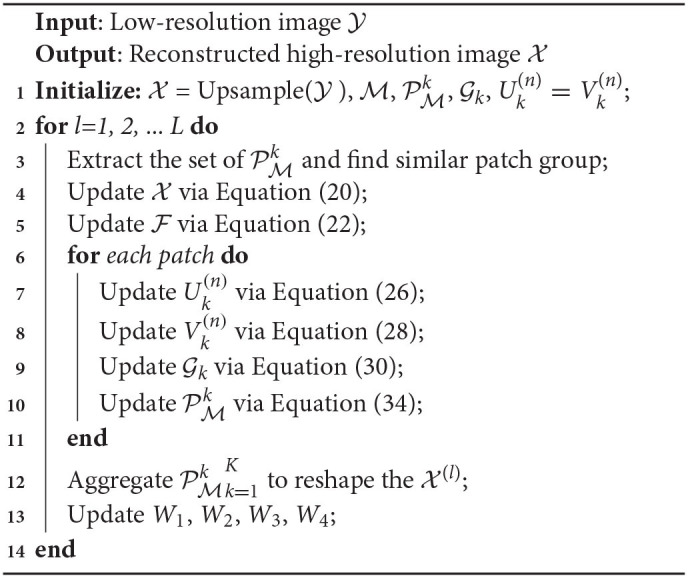
*NNTDTV*.

**Update** X. Fixing the other variables, we extract terms that contain X in Equation (18). We update X by minimizing:


(19) $$\begin{array}{l} \begin{array}{c} \underset{X}{\text{min }}||DSX-Y|{|}_{F}^{2}+\frac{w_{1}}{2}||X-M+\frac{W_{1}}{w_{1}}|{|}_{F}^{2}+\frac{w_{2}}{2}||F\\ -G_{w}X+\frac{W_{2}}{w_{2}}|{|}_{F}^{2} \end{array} \end{array}$$

The partial derivative of Equation (19) with respect to X is as follows:


(20) $$\begin{array}{l} \begin{array}{c} 2{\left(DS\right)}^{T}DSX+w_{1}X+w_{2}G_{w}^{\prime }G_{w}X=2{\left(DS\right)}^{T}Y+w_{1}M\\ \;-W_{\text{1}}+w_{2}G_{w}^{\prime }F+W_{2}G_{w}^{\prime } \end{array} \end{array}$$

where (*DS*)^*T*^ denotes the transposes of *DS* and $G_{w}^{\prime }$ denotes the adjoint of *G*_*w*_.

**Update** F. Update F by minimizing:


(21) $$\begin{array}{l} \begin{array}{c} \underset{F}{\text{min }}\lambda_{tv}||F|{|}_{1}+\frac{w_{2}}{2}||F-G_{w}X+\frac{W_{2}}{w_{2}}|{|}_{F}^{2}\\ =\underset{F}{\text{min }}\overset{N}{\underset{n=1}{\sum }}\left(\lambda_{tv}\alpha_{n}{\left||F_{\left(n\right)}|\right|}_{1}+\frac{w_{2}}{2}||F-G_{w}X+\frac{W_{2}}{w_{2}}|{|}_{F}^{2}\right) \end{array} \end{array}$$

Then, we have:


(22) $$\begin{array}{l} F\text{= fol}{\text{d}}_{n}\left[Soft_{\lambda_{tv}\alpha_{i}/w_{2}}\left(G_{w}X_{\left(n\right)}-\frac{{W_{2}}_{\left(i\right)}}{w_{2}}\right)\right] \end{array}$$

where *Soft* denotes the soft-thresholding operator, which is defined as:


(23) $${\text{Soft}}_{\Delta }(x)=\{\begin{array}{ll} x-\Delta , & if\;x>\Delta ,\\ x+\Delta , & if\;x<\Delta ,\\ 0, & otherwise. \end{array}$$

where *x* ∈ ℝ and Δ > 0.

**Update** $U_{k}^{\left(n\right)}$. Update $U_{k}^{\left(n\right)}$ by minimizing:


(24) $$\begin{array}{l} \underset{U}{\text{min }}\lambda_{1}||U_{k}^{\left(n\right)}|{|}_{P_{\lambda }}+\frac{w_{3}}{2}||U_{k}^{\left(n\right)}-V_{k}^{\left(n\right)}+\frac{W_{3\left(k\right)}^{n}}{w_{3}}|{|}_{F}^{2} \end{array}$$

To solve Equation (24), we introduce the singular value shrinkage operator, which is defined as:


(25) $$\begin{array}{l} S_{\tau }\left(X\right):=U_{X}D_{\tau }\left(\underset{X}{\sum }\right)V_{X}^{T}, \end{array}$$

where $X=U_{X}\underset{X}{\sum }V_{X}^{T}$ is the singular value decomposition of *X*. For a matrix *A*, [_*D*_τ_(*A*)]*ij*_ = sgn(*A*(*ij*)(|_*A*_*ij*_|−τ)+_. $U_{k}^{\left(n\right)}$ can be obtained by:


(26) $$\begin{array}{l} {\text{U}}_{k}^{\left(n\right)}=S_{\lambda_{1}\lambda /w_{3}}\left(V_{k}^{\left(n\right)}+\frac{W_{3\left(k\right)}^{n}}{w_{3}}\right) \end{array}$$

**Update** $V_{k}^{\left(n\right)}$. Update $V_{k}^{\left(n\right)}$ by minimizing:


(27) $$\begin{array}{l} \begin{array}{c} \underset{V}{\text{min }}\overset{N}{\underset{n=1}{\sum }}\left(\frac{w_{3}}{2}||U_{k}^{\left(n\right)}-V_{k}^{\left(n\right)}+\frac{W_{3\left(k\right)}^{n}}{w_{3}}|{|}_{F}^{2}\right)\text{+}\frac{w_{4}}{2}||P_{M}^{k}\\ -G_{k}\times_{1}V_{k}^{\left(1\right)}\times_{2}V_{k}^{\left(2\right)}\cdots \times_{N}V_{k}^{\left(N\right)}\frac{W_{4\left(k\right)}^{n}}{w_{4}}|{|}_{F}^{2} \end{array} \end{array}$$

We obtain the solution of Equation (27) as follows:


(28) $$\begin{array}{l} \begin{array}{c} V_{k}^{\left(n\right)}=\left[{\text{- W}}_{3\left(k\right)}^{n}+w_{3}U_{k}^{\left(n\right)}+\left(W_{4\left(k\right)}+w_{4}P_{M}^{k}\right)V_{k}^{\left(-n\right)}G_{\left(n\right)}^{T}\right]\\ \;\times {\left(w_{4}I+w_{4}G_{\left(n\right)}V_{k}^{\left(-n\right)T}V_{k}^{\left(-n\right)}G_{\left(n\right)}^{T}\right)}^{-1} \end{array} \end{array}$$

**Update** $G_{k}$. Update G by minimizing:


(29) $$\begin{array}{l} \begin{array}{c} \underset{G}{\text{min }}\lambda_{2}||G_{k}|{|}_{F}^{2}+\frac{w_{4}}{2}||P_{M}^{k}-G_{k}\times_{1}V_{k}^{\left(1\right)}\times_{2}V_{k}^{\left(2\right)}\cdots \times_{N}V_{k}^{\left(N\right)}\\ +\frac{W_{4\left(k\right)}}{w_{4}}|{|}_{F}^{2} \end{array} \end{array}$$

We obtain the following solution of $G_{k}$:


(30) $$\begin{array}{l} \begin{array}{c} vec\left(G\right)={\left[V^{\left(-n\right)T}V^{\left(-n\right)}⊗w_{4}V^{\left(n\right)T}V^{\left(n\right)}+\lambda_{2}I\right]}^{-1}\\ \times vec\left[V^{\left(n\right)T}\left(W_{4\left(k\right)}+w_{4}P_{M}^{k}\right)V^{\left(-n\right)}\right] \end{array} \end{array}$$

**Update** M. Fixing the other variables, we extract terms containing M in Equation (18). We update M by minimizing:


(31) $$\begin{array}{l} \begin{array}{c} \underset{M}{\text{min }}\frac{w_{1}}{2}||X-M+\frac{W_{\text{1}}}{w_{1}}|{|}_{F}^{2}\\ +\overset{K}{\underset{k=1}{\sum }}\left(\frac{w_{4}}{2}||P_{M}^{k}-G_{k}\times_{1}U_{k}^{\left(1\right)}\times_{2}U_{k}^{\left(2\right)}\cdots \times_{4}U_{k}^{\left(N\right)}+\frac{W_{4\left(k\right)}}{w_{4}}|{|}_{F}^{2}\right) \end{array} \end{array}$$

We obtain M by updating $P_{M}^{k}$ and aggregating them:


(32) $$\begin{array}{l} M=\overset{K}{\underset{k=1}{\sum }}P_{M}^{k}. \end{array}$$

Here, the formula to update $P_{M}^{k}$ can be expressed as:


(33) $$\begin{array}{l} \begin{array}{c} \underset{P_{M}^{k}}{\text{min }}\frac{w_{1}}{2}||P_{X}^{k}-P_{M}^{k}+\frac{W_{\text{1(k)}}}{w_{1}}|{|}_{F}^{2}\\ +\overset{K}{\underset{k=1}{\sum }}\left(\frac{w_{4}}{2}||P_{M}^{k}-G_{k}\times_{1}U_{k}^{\left(1\right)}\times_{2}U_{k}^{\left(2\right)}\cdots \times_{4}U_{k}^{\left(N\right)}+\frac{W_{4\left(k\right)}}{w_{4}}|{|}_{F}^{2}\right) \end{array} \end{array}$$

Then, we obtain the following closed-form solution of $P_{M}^{k}$:


(34) $$\begin{array}{l} P_{M}^{k}=\frac{w_{4}C+w_{1}P_{X}^{k}+W_{\text{1}\left(k\right)}-W_{4\left(k\right)}}{w_{4}+w_{1}} \end{array}$$

where $C=G_{k}\times_{1}U_{k}^{\left(1\right)}\times_{2}U_{k}^{\left(2\right)}\cdots \times_{4}U_{k}^{\left(N\right)}$.

## 3. Experiments

### 3.1. Database

We conduct extensive experiments to evaluate the effectiveness of our method compared with the SOTA methods. We adopt four kinds of medical images including the synthetic brain MRI data selected from the BrainWeb database (Cocosco et al., 1997)^1^, the real abdominal CT data from the NIH pancreas segmentation database (Roth et al., 2015)^2^, the real prostate MRI data from the NCI-ISBI 2013 database,^3^ and dental image published by TF-SISR.

The BrainWeb database contains a set of synthetic brain MRI data volumes (image size of 181 × 217 × 181 and spatial resolution of 1 *mm*^3^ × 1 *mm*^3^ × 1 *mm*^3^) produced by an MRI simulator. The NIH pancreas segmentation database contains 82 real contrast-enhanced abdominal CT volumes (image size of 256 × 256 × B, where B ∈ [90, 233] and spatial resolution from 0.5 *mm*^3^ × 0.5 *mm*^3^ × 0.5 *mm*^3^ to 1 *mm*^3^ × 1 *mm*^3^ × 1 *mm*^3^). The NCI-ISBI 2013 database contains 30 prostate MRI cases (image size of 384 × 384 × B, where B ∈ [113, 217] and spatial resolution from 0.6 *mm*^3^ × 0.6 *mm*^3^ × 3.6 *mm*^3^ to 0.625 *mm*^3^ × 0.625 *mm*^3^ × 4 *mm*^3^). The dental image published by the TF-SISR contains a set of dental CT data (image size of 282 × 266 × 392). The CBCT image was obtained with the Carestream 81003D system. The linewidth resolution of the CBCT machine was 500 μm and the volumes have a voxel size of 80 × 80 × 80 μ*m*^3^. The μCT image was obtained with a Quantum FX system from Perkin Elmer, with a linewidth resolution of 50 μm, and voxel size of 40 × 40 × 40 μ*m*^3^.

### 3.2. Experimental Settings

Experiments on the BrainWeb database, NIH pancreas segmentation database, NCI-ISBI 2013 database, and LR images are obtained by applying Gaussian blurring with a blur kernel of 1 voxel wide and downsampling the original HR images with a factor of 2, similar to LRTV (Shi et al., 2015), and the original HR images are used as ground truth (GT).

In the experiment with dental images published by the TF-SISR method, our method can be validated on the real-world database. We use the CBCT as input and the μCT as GT. we follow the TF-SISR setting, including the downsampling and blurring kernel type, to ensure integrity and fairness. To evaluate the reconstruction effect, we compare the reconstruction results of each method with μCT images acquired from the same sample.

**Figure 2:**
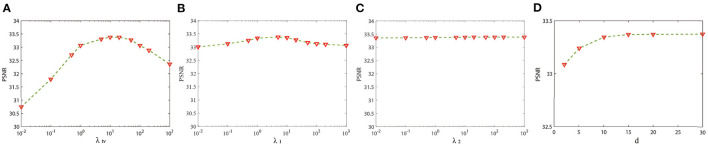
Sensitivity analysis of two regularization parameters: **(A)** The peak signal-to-noise ratio (PSNR) value vs. λ_*tv*_; **(B)** the PSNR value vs. λ_1_; **(C)** the PSNR value vs. λ_2_; **(D)** the PSNR value vs. the number of similar patches.

There are four important parameters in our model including λ_*tv*_, λ_1_, λ_2_, and d. λ_*tv*_, λ_1_, and λ_2_ control the balance of 3DTV and nonlocal low-rank regularization terms, and d is the number of similar patches that form the fourth-order tensor $P_{X}^{k}$. We select 20 serial representative slices from the BrainWeb database for parameters analysis and selection. In Figure 2, we show the relationship between PSNR and the regularization parameters λ_*tv*_, λ_1_, and λ_2_ in Equation (15) with the other parameters fixed at optimal values. The change of λ_*tv*_ has the biggest influence on PSNR of results, while the change of λ_2_ has the least influence. It can be seen that when λ_*tv*_, λ_1_, and λ_2_ vary within a wide range, PSNR can reach a high value. We observed similar behavior in other cases. The value of λ_*tv*_ and λ_1_ and λ_2_ can be chosen in [5, 20] and [1, 10], respectively. In all experiments, the parameters are set as follows: the regularization parameters λ_*tv*_, λ_1_, and λ_2_ are fixed to 10, 5, and 20, respectively. Figure 2D shows the PSNR gains vs. the number of similar patches. The PSNR of reconstruction results becomes stable when the number of patches is larger than 10. We set the number of similar patches as 15 in our experiment. Great experimental results show that the set of parameters has good universality for different types of data. For LRTV and TF-SISR, we adjust the parameters for each database to optimize experimental results.

### 3.3. Quantitative Comparison

To evaluate the performance of our method on medical images, we compare our method with the nearest neighbor (NN) interpolation method, two state-of-the-art (SOTA) methods for the medical image 3D super-resolution including the low-rank and total variation regularizations (LRTV) method (Shi et al., 2015) and the tensor factorization single image super-resolution (TF-SISR) method (Hatvani et al., 2019). In 3D medical image super-resolution methods, LRTV and TF-SISR achieve competitive results. We use two quantitative picture quality indices to evaluate the quality of the reconstructed image, i.e., the peak signal-to-noise ratio (PSNR) and the structural similarity index (SSIM). It is known that higher values of PSNR and SSIM indicate better performances.

**Figure 3:**
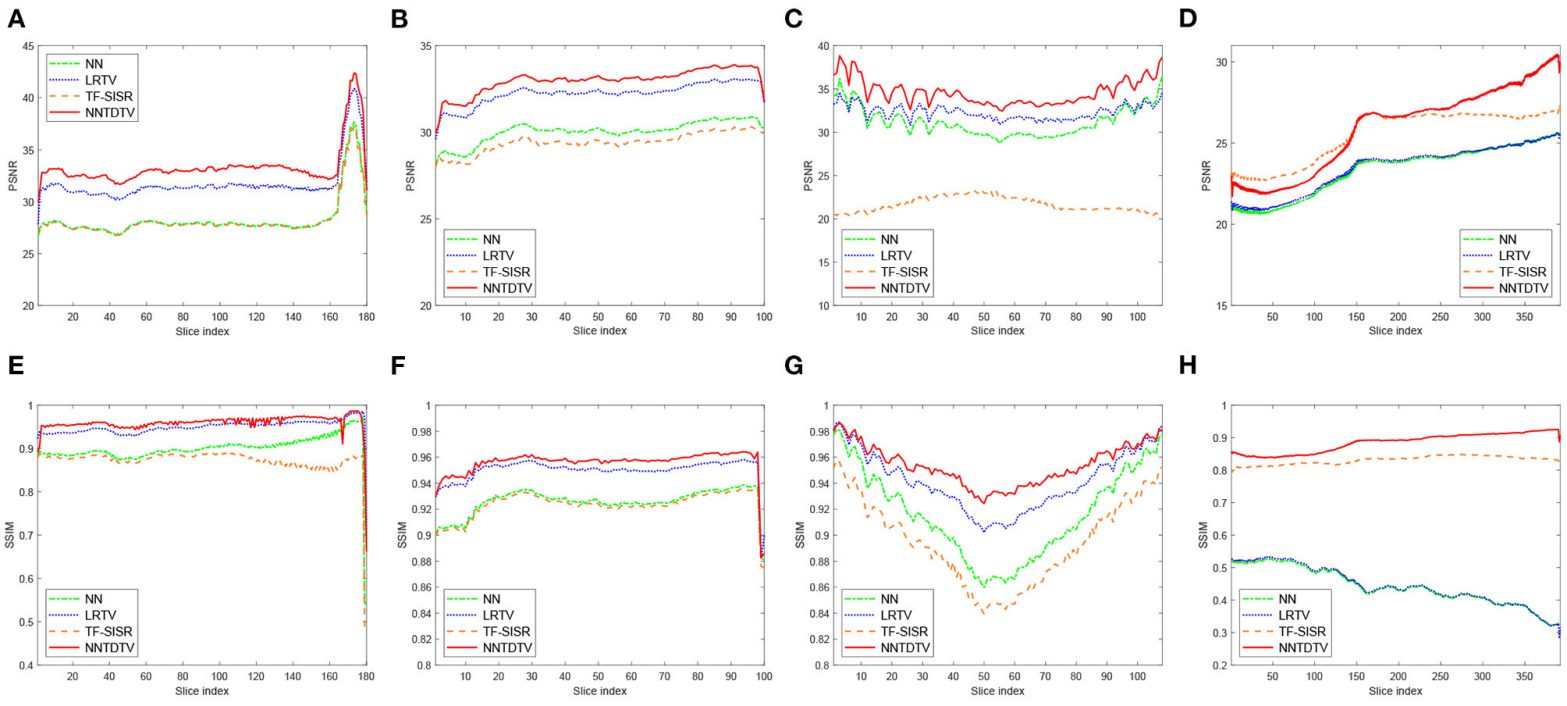
Detailed quantitative evaluation (PSNR and SSIM) of different methods for each slice: **(A,E)** The Brainweb database; **(B,F)** the NIH pancreas segmentation database; **(C,G)** the NCI-ISBI 2013 database; and **(D,H)** the dental image.

Table 1 shows the quantitative results of the BrainWeb database, NIH pancreas segmentation database, NCI-ISBI 2013 database, and dental image. The bolding values in table means the best results. These results show that our reconstruction method in terms of PSNR and SSIM outperforms the existing method in different types of medical images. Through Table 1, we can find that our model achieves the highest MPSNR value than those of other methods. The quantitative results by TF-SISR are relatively low in the first three databases. This shows that it is not enough to only use tensor factorization to recover images with complex structures, and it is necessary to add more priors. This is also demonstrated by our quantitative and visual results. Our model makes full use of the 3DTV and the nonlocal similarity to suppress noise and preserve tiny details, thus achieving the best results. For a further detailed analysis of the super-resolution results, we randomly select a set of data from the BrainWeb database, the NIH pancreas segmentation database, and the NCI-ISBI 2013 database and show the SSIM and PSNR values of each slice in Figure 3. Our method obtains much higher values of SSIM and PSNR than other methods for almost every slice.

**Table 1:** Quantitative results [peak signal-to-noise ratio (PSNR), structural 254 similarity index (SSIM)] by different methods on four medical databases.

**Database**		**NN**	**LRTV**	**TF-SISR**	**NNTDTV**
BrainWeb	PSNR (dB)	28.3336	31.7784	28.2847	**33.3313**
	SSIM	0.8996	0.9492	0.8955	**0.9593**
NIH pancreas	PSNR (dB)	30.6488	32.6053	27.2705	**33.2831**
	SSIM	0.9039	0.9242	0.8935	**0.9289**
NCI-ISBI 2013	PSNR (dB)	29.6478	31.2985	26.6026	**33.2587**
	SSIM	0.8632	0.9019	0.8582	**0.9239**
Dental	PSNR (dB)	23.3410	23.4459	25.5183	**25.9325**
	SSIM	0.4418	0.4449	0.8310	**0.8851**

*The best results are shown in bold*.

### 3.4. Visual Quality Comparison

**Figure 4:**
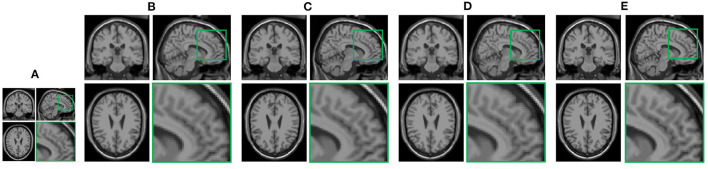
Reconstruction results and their corresponding details of synthetic brain MRI data from Brainweb database: **(A)** Low-resolution (LR) band with downsampling factor 2, **(B)** nearest neighbor (NN), **(C)** low-rank and total variation regularizations (LRTV), **(D)** TF-SISR, **(E)** nonconvex nonlocal Tucker decomposition with weighted 3D total variation (NNTDTV).

**Figure 5:**
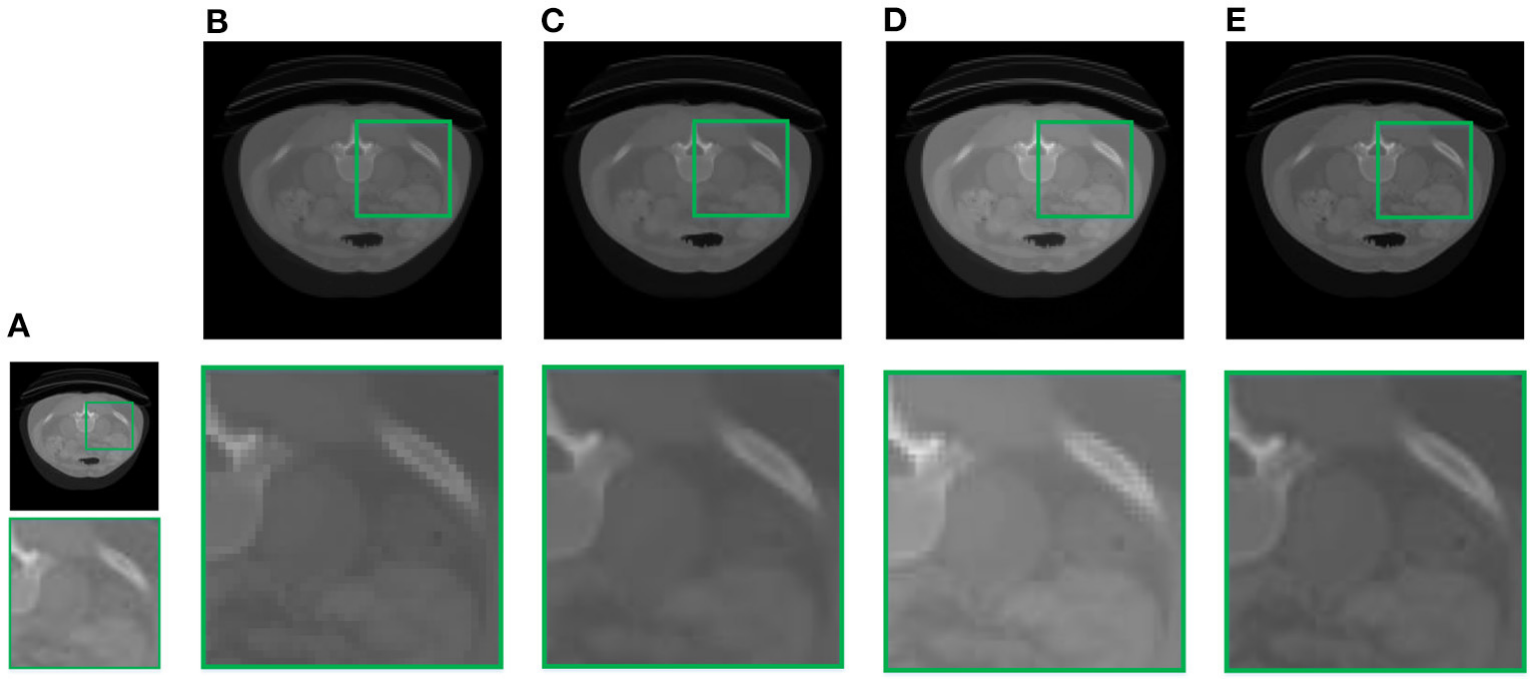
Reconstruction results and corresponding details of contrast-enhanced abdominal CT data from NIH pancreas segmentation database: **(A)** LR band with downsampling factor 2, **(B)** NN, **(C)** LRTV, **(D)** TF-SISR, **(E)** NNTDTV.

**Figure 6:**
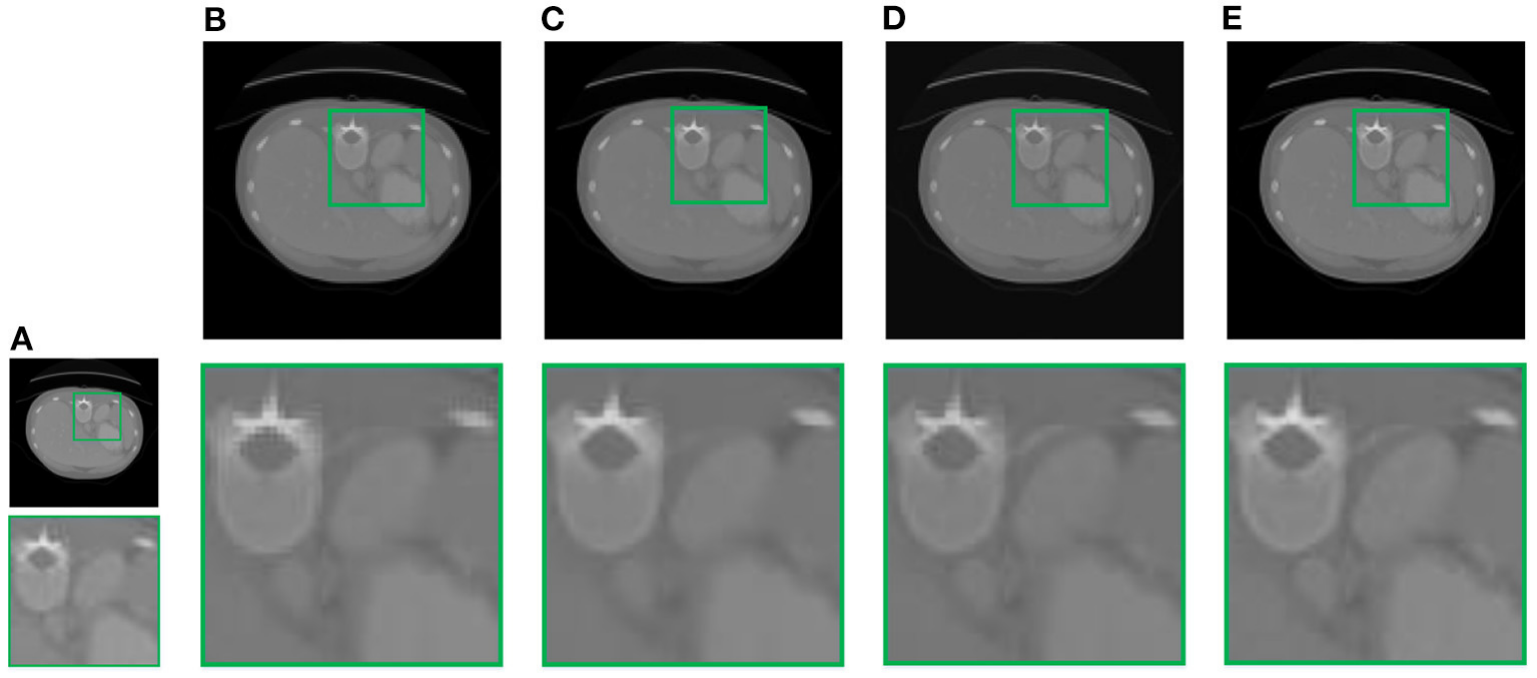
Reconstruction results and corresponding details of contrast-enhanced abdominal CT data from NIH pancreas segmentation database: **(A)** LR band with downsampling factor 2, **(B)** NN, **(C)** LRTV, **(D)** TF-SISR, **(E)** NNTDTV.

**Figure 7:**
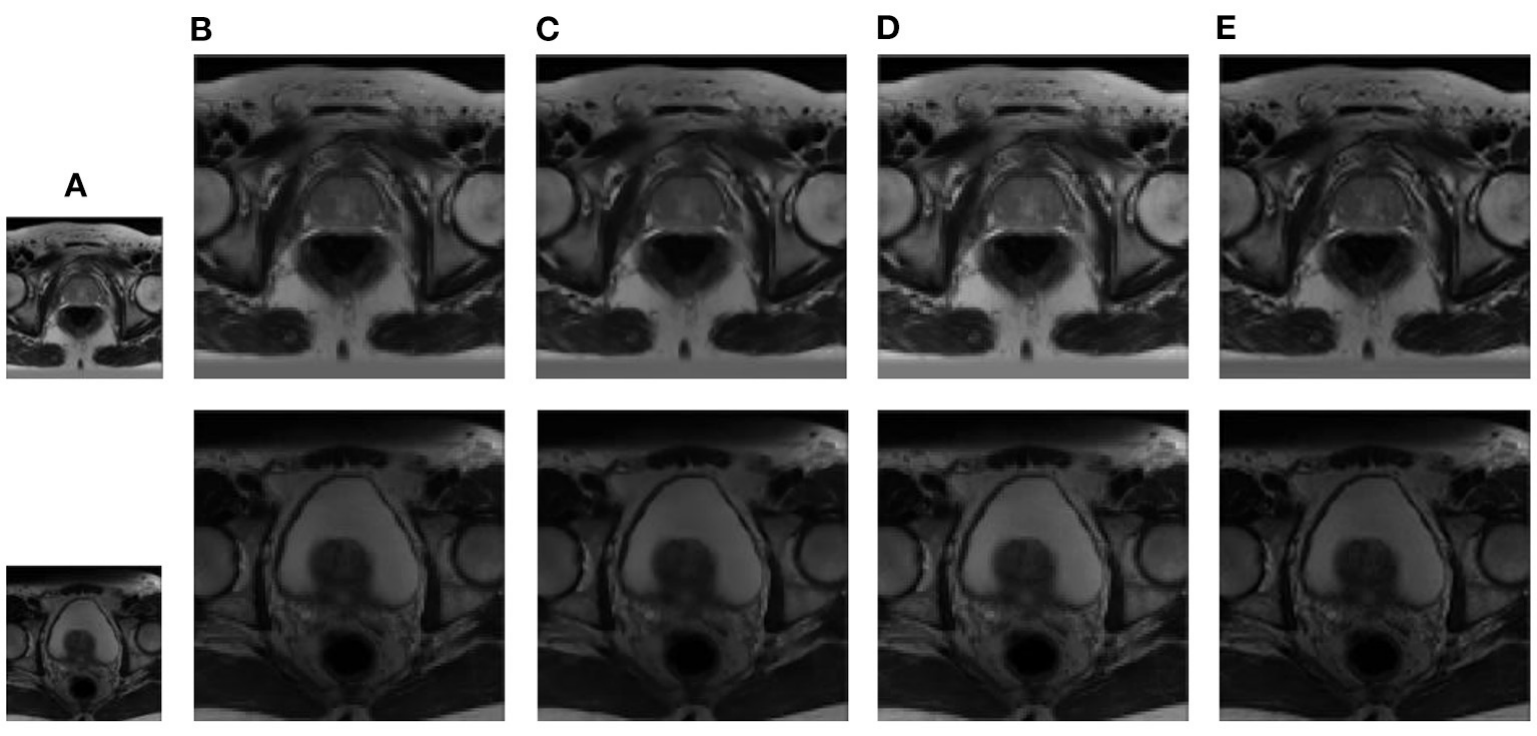
Reconstruction results of prostate MRI data from NCI-ISBI 2013 database: **(A)** LR band with downsampling factor 2, **(B)** NN, **(C)** LRTV, **(D)** TF-SISR, **(E)** NNTDTV.

In this section, we demonstrate the visual results of each method on four databases. For the results of the BrainWeb databases, a typical slice of the coronal, sagittal, and axial views is shown in Figure 4, and a zoom in of the frontal region in the sagittal view is also provided. Figures 5, 6 show a set of representative reconstruction results and the corresponding details from the NIH pancreas segmentation database, respectively. Figure 7 shows two sets of results from the NCI-ISBI 2013 database. As shown in the figures, the reconstruction results of NN and TF-SISR have obvious serrated edges while the results of LRTV are too smooth to keep the details clear. Compared with them, our method not only preserves the exact color but also reconstructs clearer edges while maintaining the local smoothness.

**Figure 8:**
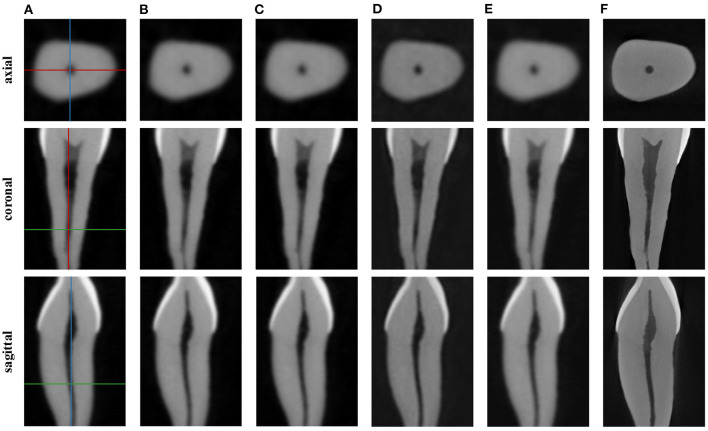
Reconstruction results of dental image: **(A)** Cone beam computed tomography (CBCT), **(B)** NN, **(C)** LRTV, **(D)** TF-SISR, **(E)** NNTDTV, **(F)** micro-CT (μCT). Each column corresponds to an axial, a coronal, and a sagittal slice. The location of the slices in the volume is illustrated on the CBCT images in colored lines.

Notably, the TF-SISR method obtains a relatively good performance only on dental images. This may be because the TF-SISR method only utilizes tensor factorization to recover images without any prior information. Therefore, when the structure of an image is complex, the reconstruction effect is not satisfactory. The TF-SISR method was proposed for 3D image super-resolution and applied to dental images. In this part of the experiment, we follow the experimental setting of the TF-SISR method. We show the PSNR and SSIM in Table 1 and the corresponding axial, coronal, and sagittal slices in Figure 8. Our method is not only superior to other methods with various types of medical images but also achieves a slightly better effect than TF-SISR with the dental image. This shows that our method has good performance and generalization.

## 4. Discussion

Super-resolution reconstruction provides an economical and effective solution to improve the resolution of CT and MRI at the software level. However, there are few super-resolution methods designed for 3D medical images. We proposed a single image super-resolution method based on tensor low-rank decomposition that combines the local smoothness and nonlocal similarity and apply to 3D CT and MRI data. Our method shows better quantitative results and visual quality compared with existing 3D medical super-resolution methods. We maintain the local smoothness while keeping the clear edges of images.

This study has certain limitations. First, nonlocal similarity prior makes full use of the structural information of images and improves the effect of super-resolution reconstruction. However, it increases computational costs. In the future study, we will explore more efficient priors to include in our framework. We will further consider the global spatial correlation in all directions of data by constructing reasonable tensors and then performing low-rank constraints. We will combine the low-rank prior data with the deep learning method to obtain accurate data features and achieve better super-resolution reconstruction results. Second, although our super-resolution model can theoretically be used for images of any dimension, we only verify it on 3D images in this article. In the future, the experiments will be conducted on diverse data.

## 5. Conclusion

In this article, we proposed a new super-resolution method based on nonlocal Tucker tensor decomposition and 3D TV regularization. Nonlocal Tucker tensor decomposition fully exploits the spatial and inter-frame low-rank information to reconstruct the data. The 3D TV regularization term retains the local smoothness of spatial and spectral information, thus enhancing the detailed information. Extensive experimental studies on four different kinds of medical data, including MRI data of the brain and prostate and CT data of the abdominal and dental, validated our method compared with the SOTA methods.

## Data Availability Statement

Publicly available datasets were analyzed in this study. This data can be found at: BrainWeb, https://brainweb.bic.mni.mcgill.ca/brainweb/.

## Ethics Statement

Ethical review and approval was not required for the study on human participants in accordance with the local legislation and institutional requirements. Written informed consent from the patients/participants or legal guardian/next of kin was not required to participate in this study in accordance with the national legislation and the institutional requirements.

## Author Contributions

XC and ZH contributed to the conception and design of the study. HJ wrote this manuscript and participated in the whole experiment process. BL wrote sections of the manuscript. All authors contributed to manuscript revision, read, and approved the submitted version.

## Funding

This study was supported in part by the National Natural Science Foundation of China under Grant 61903358, 61873259, and 61821005, in part by the Youth Innovation Promotion Association of the Chinese Academy of Sciences under Grant 2022196 and Y202051, National Science Foundation of Liaoning Province under Grant 2021-BS-023, and in part by the National Key Research and Development Program of China under Grant 2020YFB1313400.

## Conflict of Interest

The authors declare that the research was conducted in the absence of any commercial or financial relationships that could be construed as a potential conflict of interest.

## Publisher's Note

All claims expressed in this article are solely those of the authors and do not necessarily represent those of their affiliated organizations, or those of the publisher, the editors and the reviewers. Any product that may be evaluated in this article, or claim that may be made by its manufacturer, is not guaranteed or endorsed by the publisher.

## Footnotes

^1^The BrainWeb database can be downloaded at: https://brainweb.bic.mni.mcgill.ca/brainweb/.

^2^The NIH pancreas segmentation database can be downloaded at: https://wiki.cancerimagingarchive.net/display/Public/Pancreas-CT.

^3^The NCI-ISBI 2013 database can be downloaded at: https://liuquande.github.io/SAML/.
